# Nomograms Combining Ultrasonic Features With Clinical and Pathological Features for Estimation of Delphian Lymph Node Metastasis Risk in Papillary Thyroid Carcinoma

**DOI:** 10.3389/fonc.2021.792347

**Published:** 2021-12-23

**Authors:** Qi Qi, Pan Xu, Cheng Zhang, Suping Guo, Xingzhi Huang, Songli Chen, Yaohui Li, Aiyun Zhou

**Affiliations:** Department of Ultrasonography, The First Affiliated Hospital of Nanchang University, Nanchang, China

**Keywords:** Delphian lymph nodes metastasis, nomograms, ultrasound, papillary thyroid carcinoma, risk factors

## Abstract

**Background:**

This work explores the clinical significance of Delphian lymph nodes (DLN) in thyroid papillary carcinoma (PTC). At the same time, a nomogram is constructed based on clinical, pathological, and ultrasonic (US) features to evaluate the possibility of DLN metastasis (DLNM) in PTC patients. This is the first study to predict DLNM using US characteristics.

**Methods:**

A total of 485 patients, surgically diagnosed with PTC between February 2017 and June 2021, all of whom underwent thyroidectomy, were included in the study. Using the clinical, pathological, and US information of patients, the related factors of DLNM were retrospectively analyzed. The risk factors associated with DLNM were identified through univariate and multivariate analyses. According to clinical + pathology, clinical + US, and clinical + US + pathology, the predictive nomogram for DLNM was established and validated.

**Results:**

Of the 485 patients with DLN, 98 (20.2%) exhibited DLNM. The DLNM positive group had higher positive rates of central lymph node metastasis (CLNM), lateral lymph node metastasis (LLNM), and T3b–T4b thyroid tumors than the negative rates. The number of CLNM and LLNM lymph nodes in the DLNM+ group was higher as compared to that in the DLNM- group. Multivariate analysis demonstrated that the common independent risk factors of the three prediction models were male, bilaterality, and located in the isthmus. Age ≥45 years, located in the lower pole, and nodural goiter were protective factors. In addition, the independent risk factors were classified as follows: (I) P-extrathyroidal extension (ETE) and CLNM based on clinical + pathological characteristics; (II) US-ETE and US-CLNM based on clinical + US characteristics; and (III) US-ETE and CLNM based on clinical +US + pathological features. Better diagnostic efficacy was reported with clinical + pathology + US diagnostic model than that of clinical + pathology diagnostic model (AUC 0.872 vs. 0.821, *p* = 0.039). However, there was no significant difference between clinical + pathology + US diagnostic model and clinical + US diagnostic model (AUC 0.872 vs. 0.821, *p* = 0.724).

**Conclusions:**

This study found that DLNM may be a sign that PTC is more invasive and has extensive lymph node metastasis. By exploring the clinical, pathology, and US characteristics of PTC progression to DLNM, three prediction nomograms, established according to different combinations of features, can be used in different situations to evaluate the transfer risk of DLN.

## Introduction

Papillary thyroid carcinoma (PTC) is the most common histological type of thyroid malignancy, prone to early lymph node metastasis. Although it has an ideal prognosis, lymph node metastasis increases the risk of long-term recurrence (1, 2). The most common metastatic site of PTC is the central cervical lymph node (CLN), followed by lateral cervical lymph nodes (LLN) (3, 4). Cases of jumping metastasis are rare. Paratracheal lymph nodes, anterior tracheal lymph nodes, and Delphian lymph node (DLN) are, in most cases, associated with CLN. In surgical management of thyroid papillary carcinoma, clinicians are needed to judge the presence of lymph node metastasis according to preoperative imaging examination and intraoperative frozen pathological results of lymph nodes. It is difficult to explore preoperative imaging because the DLN position is concealed.

DLN, also known as prelaryngeal lymph node or precricoid lymph node, is a group of lymph nodes located in front of the cricoid membrane between cricoid cartilage and thyroid cartilage. It mainly receives lymphatic drainage from the throat and thyroid. Studies have revealed a positive correlation of DLN metastasis (DLNM) with a poor prognosis due to extensive regional lymph node metastasis, recurrence, and death of head and neck tumors (especially laryngeal cancer) (5–12). Although there are few relevant studies on the metastasis of PTC to DLN, some studies have recently demonstrated the potential association of DLNM with central lymph node metastasis (CLNM) and lateral lymph node metastasis (LLNM), increasing the recurrence risk of PTC (8, 10, 11, 13–15).

Some researchers have reported clinical risk factors for DLNM in PTC, though the results vary across studies. None of the studies has included ultrasonic (US) features in preoperative analysis. US examination is the most used and successful imaging approach for thyroid nodules. US allows for vivid evaluation of the morphological characteristics of thyroid nodules and cervical lymph nodes. Therefore, the present study aims to explore the significance of DLN in PTC invasion and metastasis. Then, establish multiple nomographic prediction models based on clinical, pathological, and US characteristics to predict the risk of DLNM. The findings aims to enrich the clinicians with a preoperative understanding of the condition and guide clinical decision-making to reduce the recurrence and metastasis probability of PTC.

## Materials and Methods

### Patients

This is a retrospective study. The treatment records of 1,279 PTC patients who underwent the first thyroidectomy in the First Affiliated Hospital of Nanchang University between February 2017 and June 2021 were reviewed. All patients underwent preoperative thyroid ultrasonography. DLN was detected in 485 (37.9%) patients during operation. The clinical, US, and pathological information of the 485 patients were collected, and the patients were divided randomly into the training cohort (388 patients, 80%) and the validation cohort (97 patients, 20%). The authors are accountable for all aspects of the work in ensuring that questions related to the accuracy or integrity of any part of the work are appropriately investigated and resolved. All procedures performed in this study involving human participants were in accordance with the Declaration of Helsinki (as revised in 2013).

### US Check

Three radiologists (SG, PX, and CZ) with 5–10 years of experience in ultrasonic diagnosis of thyroid, performed US image acquisition using Philips iu22 and Philips EPIQ7 (US system, Philips Medical System, Bothell, WA, USA) US diagnostic instrument. The linear probe was 5–12 MHz, with the condition set to “thyroid” mode.

Three radiologists explored the thyroid region of the 1,279 patients. The complete thyroid US images were loaded into the US system, and clear nodule images were obtained. The position of the nodule (right/left lobe, upper/middle/lower, full, and isthmus) was recorded, and measurements of the vertical and horizontal diameters of the nodule were taken. The maximum diameter of the nodule and the presence of suspected cervical lymph node metastasis were recorded. After review of 1,279 cases, the thyroid US image features of 485 patients with DLN were independently recorded by two radiologists (PX and AZ) with more than 8 years of experience in US diagnosis of thyroid. The two reviewers compared and discussed their results in case of dispute to reach a consensus. The US image features recorded in this study include the following: nodule size (maximum diameter of nodule), nodule location (isthmus, upper/middle/lower, full), nodule composition (solid or non-solid), nodule echo (hypoechoic, very hypoechoic, non-hypoechoic), nodule shape (regular, irregular), nodule margin (smooth, ill-defined), aspect ratio (A/T, in cross-section/longitudinal section, longitudinal diameter of nodule divided by transverse diameter), microcalcification, multifocality, bilaterality, US-extrathyroidal extension (US-ETE), US-CLNM, nodular thyroid, and Hashimoto thyroiditis (HT). Lobulated/microneedle nodules were classified as “irregularly shaped”; A/T was classified as >1 and ≤1; the presence of calcifications ≤1 mm within the nodule was classified as “microcalcifications”; the presence of multiple PTCs in the entire thyroid gland was considered “multifocality”; here, the nodule with the largest diameter was recorded first; the presence of PTCs in both thyroid lobes was classified as “bilaterality”. The diagnosis of “multifocality”, “bilaterality”, and “nodular goiter” was according to ultrasonography and pathology. According to guidelines by the Joint Commission on Ultrasound for Cancer (15), US-ETE was defined according to at least one of the following US features: contact between the thyroid capsule and >25% of the circumference of the lesion; loss of the envelope line at the contact site; and tumor beyond the thyroid envelope, invading the larynx, trachea, esophagus, recurrent laryngeal nerves, common carotid artery, mediastinal vessels, and subcutaneous soft tissues. US-CLNM exhibited the following features: internal microcalcifications, hyperechoic change, round shape, vascularity, loss of hilar echogenicity, size > 5 mm, or necrosis. HT was characterized based on US images and thyroid autoantibodies.

### Surgery

Total thyroidectomy with bilaterality central node dissection (CND) was performed for PTCs present in both thyroid lobes. Besides, unilateral lobectomy with isthmus resection or total thyroidectomy with unilateral or bilaterality CND was performed for unilateral lobar PTC. Total thyroidectomy was considered for patients with unilateral PTC but specifically to those who met one or more of the following conditions: tumor size >4 cm, multiple tumors in a single lobe, Pathological-ETE (P-ETE) (referring to the invasion of the primary tumor into adjacent tissues beyond the gland), or distant metastases. Lymph node located anterior to the thyroid membrane above the thyroid isthmus and between the cricothyroid muscle was labeled as DLN. Any evidence of LLNM on preoperative examination necessitated lateral lymph node dissection (LLND).

### Statistical Analyses

All statistical data were analyzed in SPSS version 26.0 (IBM Corporation, Armonk, NY, USA) or R (version 4.1.0; http://www.r-project.org). Continuous variables were expressed as mean ± standard deviation, whereas categorical variables were expressed as number [percent (%)]. Categorical variables were compared by the *χ* ^2^ test or Fisher’s exact test. ICC (intraclass correlation coefficient) was used to evaluate inter-observer agreement. Factors independently associated with DLNM and CLNM were established *via* logistic regression. Three nomograms were established according to the binary logistic regression results to evaluate risk of DLNM preoperatively. The diagnostic efficacy was evaluated using the receiver operating characteristic (ROC) curves. The ROC curves were compared by the DeLong test. The calibration curves were used to determine the prediction compliance. The clinical application value of diagnostic models was evaluated by the decision curve analysis (DCA). A *p* < 0.05 represented a statistically significant difference.

## Results

### Patients

A total of 485 patients with DLN (gender: 113 males and 372 females; mean age: 42.4 ± 11.04 years) were analyzed in this study. Patients with DLNM were 98 (20.2%). Patients with CLNM were 233 (48.0%). A total of 87 patients were found with LLNM, of which 25 (5.2%) did not have CLNM. Detailed data are shown in **Table 1**. All the 485 subjects were divided randomly into a training cohort and a validation cohort, 388 (80%) patients and 97 (20%) patients, respectively.

**Table 1:** Comparison of clinical, ultrasound, and pathological features between all DLNM+ and DLNM- patients.

Variables	DLNM (-)	DLNM (+)	OR (95% CI)	*p*-value
Gender (%)				
Male	63 (16.3)	48 (49.0)	1 (reference)	
Female	324 (83.7)	50 (51.0)	5.357 (3.317-8.651)	<0.001
Age (%)				
<45	197 (50.9)	73 (74.5)	1 (reference)	
≥45	190 (49.1)	25 (25.5)	0.355 (0.216-0.583)	<0.001
Location (%)				
Upper	77 (19.9)	30 (30.6)	1 (reference)	0.001
Middle	194 (50.1)	54 (55.1)	0.350 (0.113-1.086)	0.069
Lower	87 (22.5)	3 (3.1)	0.278 (0.094-0.828)	0.021
Isthmus	22 (5.7)	4 (4.1)	0.048 (0.011-0.203)	<0.001
Full	7 (1.8)	7 (7.1)	0.227 (0.054-0.948)	0.042
Hashimoto thyroiditis (%)				
Negative	264 (68.2)	61 (62.2)	1 (reference)	
Positive	123 (31.8)	37 (37.8)	0.768 (0.484-1.218)	0.262
Multifocality (%)				
No	216 (55.8)	34 (34.7)	1 (reference)	
Yes	171 (44.2)	64 (65.3)	2.378 (1.498-3.773)	<0.001
Bilaterality (%)				
No	267 (69.1)	49 (50.0)	1 (reference)	
Yes	120 (30.9)	49 (50.0)	2.225 (1.418-3.492)	<0.001
Tumor size (cm) (%)				
<1.3	232 (59.9)	44 (44.9)	1 (reference)	
≥1.3	155 (40.1)	54 (54.1)	1.837 (1.175-2.872)	0.008
Shape (%)				
Regular	135 (34.9)	26 (26.5)	1 (reference)	
Irregular	252 (65.1)	72 (73.5)	1.535 (0.936-2.516)	0.032
Margin (%)				
Smooth	237 (61.2)	60 (61.2)	1 (reference)	
Ill-defined	150 (38.8)	38 (38.8)	1.001 (0.635-1.577)	0.998
A/T (%)				
≤1	303 (78.3)	83 (84.7)	1 (reference)	
>1	84 (21.7)	15 (15.3)	0.652 (0.358-1.189)	0.163
Microcalcification (%)				
No	148 (38.2)	19 (19.4)	1 (reference)	
Yes	239 (61.8)	79 (80.6)	1.371 (1.144-1.642)	0.001
US-ETE (%)				
No	287 (74.2)	30 (30.6)	1 (reference)	
Yes	100 (25.8)	68 (69.4)	6.505 (4.001-10.578)	<0.001
P-ETE (%)				
No	316 (81.7)	55 (56.1)	1 (reference)	
Yes	71 (28.3)	43 (43.9)	3.480 (2.164-5.594)	<0.001
Nodular goiter (%)				
No	232 (59.9)	83 (84.7)	1 (reference)	
Yes	155 (40.1)	15 (15.3)	0.271 (0.151-0.486)	<0.001
US-CLNM (%)				
No	329 (85.0)	64 (65.3)	1 (reference)	
Yes	58 (15.0)	34 (34.7)	3.013 (1.826-4.973)	<0.001
CLNM (%)				
No	224 (57.9)	28 (28.6)	1 (reference)	
Yes	163 (42.1)	70 (71.4)	3.436 (2.121-5.566)	<0.001
No. of CLNM	3.49 ± 2.36	5.24 ± 4.64	-	0.004
LLNM (%)				
No	38 (45.2)	12 (22.6)	-	
Yes	46 (54.8)	41 (77.4)		0.007
No. of LLNM	4.63 ± 3.00	6.59 ± 4.69	-	0.026
T3b-T4b (%)				
Negative	359 (92.8)	76 (77.6)	1 (reference)	
Positive	28 (7.2)	22 (22.4)	3.711 (2.015-6.836)	<0.001

DLNM, Delphian lymph node metastasis; A/T, aspect ratio; US-ETE, ultrasonic-based extrathyroidal extension; P-ETE, Pathology-based extrathyroidal extension; US-CLNM, ultrasonic-based central lymph node metastasis; CLNM, central lymph node metastasis; LLNM, lateral lymph node metastasis.

### DLNM+ Group *vs*. DLNM- Group

The clinical, US, and pathological characteristics of the DLNM+ group vs. the DLNM- group are outlined in **Table 1**. The DLNM+ group had a greater proportion of males and the subjects were younger (all *p* < 0.001). Larger PTC size was reported in the DLNM+ group (*p* = 0.008). Patients in the DLNM+ group were more likely to exhibit multifocality, bilaterality, P-ETE, and T3b–T4b grading (all *p* < 0.001). A higher incidence of nodular goiter was revealed in the DLNM- group (*p* < 0.001). Regarding US features, the DLNM+ group was more likely to develop PTCs characterized by irregular shape, microcalcifications, and US-ETE (*p* = 0.043, *p* < 0.001, and *p* < 0.001, respectively). Regarding LNM, the DLNM+ group exhibited a higher incidence of CLNM, US-CLNM, and LLNM than in the DLNM- group (*p* < 0.001, *p* < 0.001, and *p* = 0.007, respectively). For CLNM, US-CLNM demonstrated 34.3% sensitivity, 95.2% specificity, a positive predictive value of 87.0%, a negative predictive value of 61.1%, and 66.0% accuracy. No significant differences were observed in Hashimoto, Margin, and A/T between the two groups (all *p* > 0.05). Additionally, distant metastases were present in 6 patients (4 patients with DLNM and 2 patients without DLNM).

### Correlation Between DLN, and CLN, LLN, or T3b–T4b

The DLNM+ group registered a higher incidence of CLNM positivity as compared to that of CLNM negativity; (30.0% vs. 11.1%, *p* < 0.001). The number of CLNMs was higher in the DLNM+ group than in the DLNM- group (5.24 ± 4.64 vs. 3.49 ± 2.36, *p* = 0.004). Notably, 137 patients underwent LLND. The incidence of LLNM positivity was higher than LLNM negativity (47.1% vs. 24.0%, *p* = 0.007) in the DLNM+ group. More LLNM was reported in the DLNM+ group than in the DLNM- group (6.59 ± 4.69 vs. 4.63 ± 3.00, *p* = 0.026). Among 485 patients, a higher probability of DLNM was reported in T3b–T4b thyroid tumors than in non-T3b–T4b thyroid tumors (44.0% vs. 17.5%, *p* < 0.001). DLNM+ patients had a higher incidence of CLNM, LLNM, and T3b–T4b than DLNM- patients (71.4% vs. 42.1%, 77.4% vs. 54.8%, 22.4% vs. 7.2%) (**Table 1**).

### Inter‐Observer Agreement

Consistency analysis was conducted for the original data of the two doctors. The ICC value of each US feature was as follows: 0.878 (echogenicity), 0.972 (aspect ratio), 0.893 (microcalcification), 0.954 (margin), 0.957 (shape), 0.922 (location), and 0.956 (US-ETE) with good repeatability (all *p* < 0.001).

### Univariate Analysis

Gender (male, *p* < 0.001), age (<45, *p* < 0.001), location (Lower, 0.039; Isthmus, *p* < 0.001), multiple (*p* < 0.001), bilaterality (*p* < 0.001), PTC size (>1.3 cm, *p* = 0.013), shape (Irregular, *p* = 0.049), microcalcification (*p* = 0.002), US-ETE (*p* < 0.001), P-ETE (*p* < 0.001), no nodular goiter (*p* = 0.002), US-CLNM (*p* < 0.001), and CLNM (*p* < 0.001) exhibited a significant association with DLNM (**Table 2**).

**Table 2:** Univariate analysis of clinical, ultrasound, and pathological characteristics of the training group for DLNM prediction.

Variables	OR (95% CI)	*p*-value
Gender		
Male	1 (reference)	
Female	5.137 (3.018-8.745)	<0.001
Age		
<45	1 (reference)	
≥45	0.308 (0.176-0.540)	<0.001
Location		
Upper	1 (reference)	
Middle	0.367 (0.107-1.258)	0.111
Lower	0.288 (0.089-0.938)	0.039
Isthmus	0.058 (0.013-0.264)	<0.001
Full	0.250 (0.052-1.208)	0.085
Hashimoto thyroiditis		
Negative	1 (reference)	
Positive	1.210 (0.729-2.008)	0.460
Multifocality		
No	1 (reference)	
Yes	2.577 (1.546-4.295)	<0.001
Bilaterality		
No	1 (reference)	
Yes	2.428 (1.471-4.007)	<0.001
Tumor size (cm)		
<1.3	1 (reference)	
≥1.3	1.870 (1.141-3.065)	0.013
Shape		
Regular	1 (reference)	
Irregular	1.735 (1.003-3.001)	0.049
Margin		
Smooth	1 (reference)	
Ill-defined	0.804 (0.458-1.413)	0.448
A/T		
≤1	1 (reference)	
>1	0.988 (0.523-1.865)	0.970
Microcalcification		
No	1 (reference)	
Yes	1.364 (1.119-1.662)	0.002
US-ETE		
No	1 (reference)	
Yes	7.040 (4.284-12.795)	<0.001
P-ETE		
No	1 (reference)	
Yes	3.911 (2.293-6.670)	<0.001
Nodular goiter		
No	1 (reference)	
Yes	0.380 (0.204-0.707)	0.002
US-CLNM		
No	1 (reference)	
Yes	3.618 (2.065-6.339)	<0.001
CLNM		
No	1 (reference)	
Yes	4.274 (2.474-7.385)	<0.001

A/T, aspect ratio; US-ETE, ultrasonic-based extrathyroidal extension; P-ETE, Pathology-based extrathyroidal extension; US-CLNM, ultrasonic-based central lymph node metastasis; CLNM, central lymph node metastasis.

### Multifactorial Analysis

Multifactorial logistic regression analysis was performed according to clinical + pathological features (I), clinical + US features (II), and clinical + US + pathological features (III), respectively.

**Figure 1:**
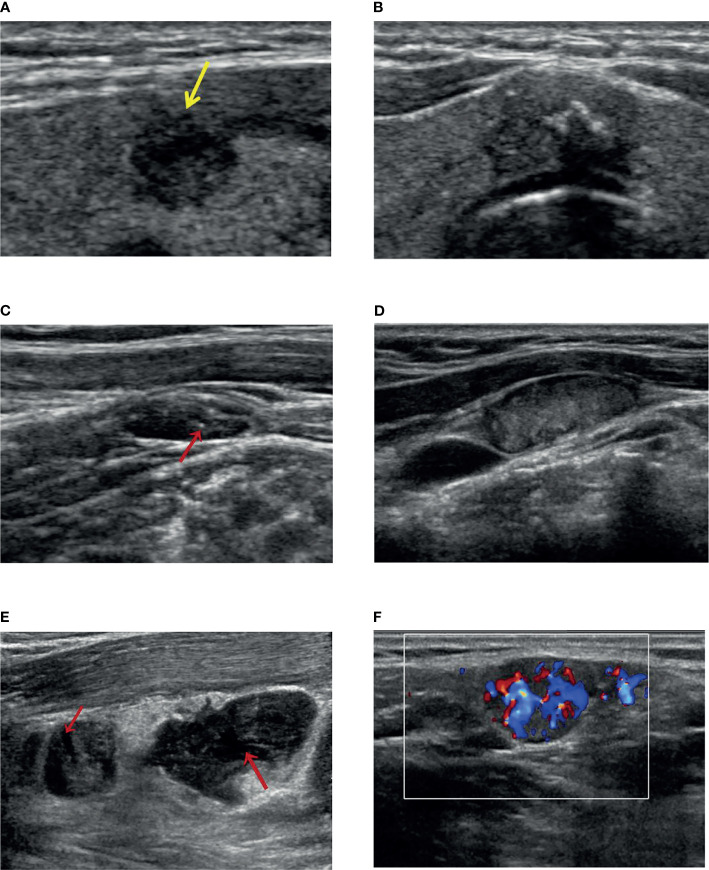
The ultrasound features for Delphian lymph node metastasis prediction. **(A)** A solid and hypoechoic thyroid nodule with an irregular shape (burr visible at the edge of the nodule) and US-ETE (yellow arrow: interrupted envelope echogenicity at the site of contact between the nodule and the envelope). **(B)** A thyroid gland located in the isthmus, with solid hypoechoic, irregular shape (lobulated), clear envelope lines, and US-ETE (the nodule is in contact with the envelope for more than 20% of its circumference). **(C)** A US-CLNM with internal microcalcification (indicated by the red arrow). **(D)** A US-CLNM with hyperechoic change and loss of hilar echogenicity. **(E)** Two US-CLNMs lost hilar echogenicity with liquefaction necrosis (indicated by the red arrow). **(F)** A US-CLNM with abundant and disordered blood flow signals.

- Analysis of clinical + pathological features demonstrated that male (OR = 3.198, 95% CI, 1.674–6.110; *p* < 0.001), bilaterality (OR = 2.195, 95% CI, 1.141–4.223; *p* = 0.019), P-ETE (OR = 2.517, 95% CI, 1.201–5.275; *p* = 0.014), CLNM (OR = 3.088, 95% CI, 1.616–5.902; *p* = 0.001), and Isthmus in PTC location (OR = 0.032, 95% CI. 0.005–0.216; *p* < 0.001) were potential independent risk factors for DLNM in PTC patients. Age ≥45 years (OR = 0.201, 95% CI, 0.099–0.408; *p* < 0.001), lower in PTC location (OR = 0.198, 95% CI, 0.041–0.969; *p* = 0.046), and nodular goiter (OR = 0.483, 95% CI, 0.235–0.990; *p* = 0.047) were potential independent protective factors for DLNM in patients with PTC (**Table 3**).
- Analysis of clinical + US features demonstrated that male (OR = 4.903, 95% CI, 2.345–10.250; *p* < 0.001), bilaterality (OR = 3.607, 95% CI, 1.711–7.604; *p* = 0.001), shape Irregular (OR = 3.082, 95% CI, 1.371–6.931; *p* = 0.006), US-ETE (OR = 8.746, 95% CI, 4.220–18.127; *p* < 0.001), US-CLNM (OR = 2.752, 95% CI, 1.221–6.202; *p* = 0.015), and Isthmus in PTC location (OR = 0.037, 95% CI, 0.005–0.273; *p* = 0.001) were potential independent risk factors for DLNM in PTC patients. Age ≥45 years (OR = 0.129, 95% CI, 0.058–0.287; *p* < 0.001), lower in PTC location (OR = 0.173, 95% CI, 0.031–0.959; *p* = 0.045), and nodular goiter (OR = 0.443, 95% CI, 0.197–0.994; *p* = 0.048) were potential independent protective factors for DLNM in patients with PTC (**Table 3** and **Figure 1**).
- Analysis of clinical + US + pathology features demonstrated that male (OR = 4.820, 95% CI, 2.299–10.104; *p* < 0.001), bilaterality (OR = 3.970, 95% CI, 1.852–8.509; *p* < 0.001), shape Irregular (OR = 3.003, 95% CI, 1.325–6.805; *p* = 0.008), US-ETE (OR = 8.826, 95% CI, 4.283–18.187; *p* < 0.001), CLNM (OR = 2.832, 95% CI, 1.409–5.693; *p* = 0.003), and Isthmus in PTC location (OR = 0.031, 95% CI, 0.004–0.232; *p* = 0.001) were potential independent risk factors for DLNM in PTC patients. Age ≥45 years (OR = 0.168, 95% CI, 0.078–0.360; *p* < 0.001), lower in PTC location (OR = 0.154, 95% CI, 0.027–0.880; *p* = 0.035), and nodular goiter (OR = 0.403, 95% CI, 0.179–0.909; *p* = 0.028) were potential independent protective factors for DLNM in patients with PTC (**Table 3**).

**Table 3:** Multivariate analysis of clinical, ultrasound, and pathological characteristics of the training group for DLNM prediction.

Variable	Clinical + Pathology		Clinical + US			Clinical + Pathology + US	
	OR (95% CI)	*p*-value	OR (95% CI)	*p*-value		OR (95% CI)	*p*-value
Gender							
Male	1 (reference)		1 (reference)			1 (reference)	
Female	3.198 (1.674-6.110)	<0.001	4.903 (2.345-10.250)	<0.001		4.820 (2.299-10.104)	<0.001
Age							
<45	1 (reference)		1 (reference)			1 (reference)	
≥45	0.201 (0.099-0.408)	<0.001	0.129 (0.058-0.287)	<0.001		0.168 (0.078-0.360)	<0.001
Location		0.005					0.009
Upper	1 (reference)		1 (reference)			1 (reference)	
Middle	0.203 (0.039-1.067)	0.060	0.228 (0.044-1.177)	0.078		0.031 (0.004-0.232)	0.070
Lower	0.198 (0.041-0.969)	0.046	0.173 (0.031-0.959)	0.045		0.154 (0.027-0.880)	0.035
Isthmus	0.032 (0.005-0.216)	<0.001	0.037 (0.005-0.273)	0.001		0.031 (0.004-0.232)	0.001
Full	0.292 (0.043-1.975)	0.207	0.225 (0.031-1.621)	0.139		0.160 (0.021-10211)	0.076
Multifocality							
No	1 (reference)		1 (reference)			1 (reference)	
Yes	-	0.354	-	0.225		-	0.309
Bilaterality							
No	1 (reference)		1 (reference)			1 (reference)	
Yes	2.195 (1.141-4.223)	0.019	3.607 (1.711-7.604)	0.001		3.970 (1.852-8.509)	<0.001
Tumor size							
<1.3	1 (reference)		1 (reference)			1 (reference)	
≥1.3	-	0.385	-	0.433		-	0.594
Shape							
Regular	-		1 (reference)			1 (reference)	
Irregular	-		3.082 (1.371-6.931)	0.006		3.003 (1.325-6.805)	0.008
Microcalcification							
No	-		1 (reference)			1 (reference)	
Yes	-		-	0.216		-	0.591
US-ETE							
No	-		1 (reference)			1 (reference)	
Yes	-		8.746 (4.220-18.127)	<0.001		8.826 (4.283-18.187)	<0.001
P-ETE							
No	1 (reference)		-			1 (reference)	
Yes	2.517 (1.201-5.275)	0.014	-			-	0.832
Nodular goiter							
No	1 (reference)		1 (reference)			1 (reference)	
Yes	0.483 (0.235-0.990)	0.047	0.443 (0.197-0.994)	0.048		0.403 (0.179-0.909)	0.028
US-CLNM							
No	-		1 (reference)			1 (reference)	
Yes	-		2.752 (1.221-6.202)	0.015		-	0.115
CLNM							
No	1 (reference)		-			1 (reference)	
Yes	3.088 (1.616-5.902)	0.001	-			2.832 (1.409-5.693)	0.003

US-ETE, ultrasonic-based extrathyroidal extension; P-ETE, Pathology-based extrathyroidal extension; US-CLNM, ultrasonic-based central lymph node metastasis; CLNM, central lymph node metastasis.

### Construction of Nomogram

We used multiple logistic regression models to build DLNM prediction models based on clinical + pathological features (I), clinical + US features (II), and clinical + US + pathological features (III), respectively, and draw interactive nomograms. The study subjects were divided randomly into a training group (388 cases, 80%) and a validation group (97 cases, 20%). The training data were used to construct the diagnostic models. The model performance was evaluated using AUC from the validation group. Each factor corresponded to a specific score, and then the value of all the scores were added to obtain the DLNM rate.

**Figure 2:**
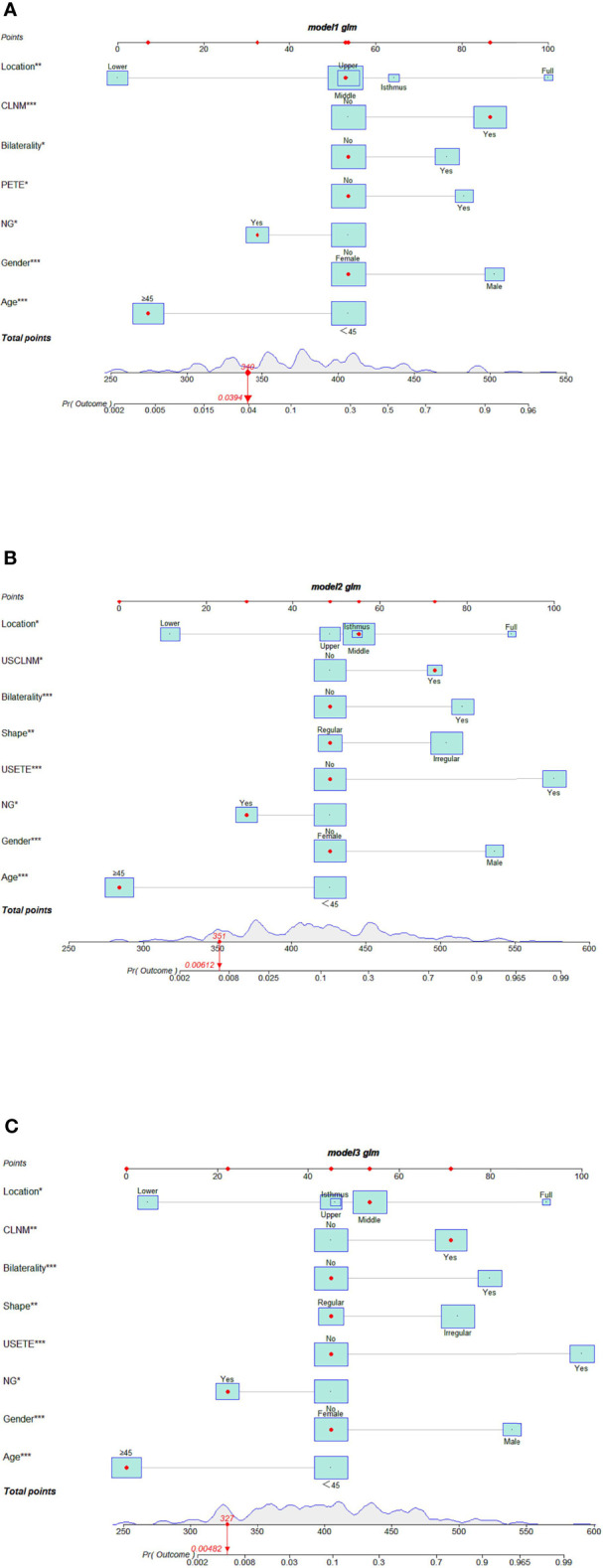
**(A)** Interactive nomogram for predicting DLNM based on clinical + pathological features. **(B)** Interactive nomogram for predicting DLNM based on clinical +US features. **(C)** Interactive nomogram for predicting DLNM based on clinical + US + pathological features. (The scores corresponding to the features in each nomogram are shown in the **Supplementary Material**. USETE, ultrasonic based extrathyroidal extension; PETE, Pathology-based extrathyroidal extension; USCLNM, ultrasonic-based central lymph node metastasis; CLNM, central lymph node metastasis; NG, Nodular goiter.

**Figure 3:**
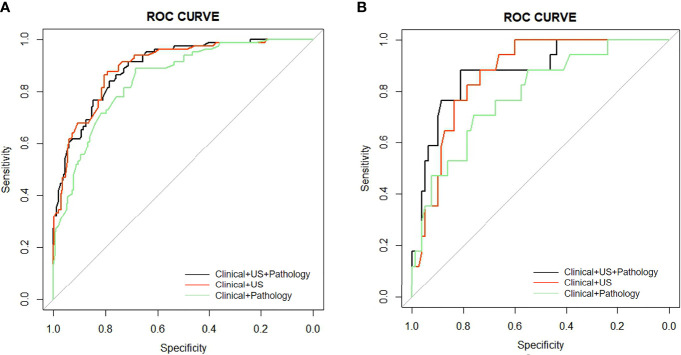
**(A)** AUC comparison of the ROC curves of the three models for predicting DLNM in the training group. **(B)** AUC comparison of the ROC curves of the three models for predicting DLNM in the validation group.

- Seven risk factors were included in the nomogram (**Figure 2A**) designed using the clinical + pathological features: gender, age, PTC location, bilaterality, P-ETE, nodular goiter, and CLNM. Full PTC contributed the most to the prediction model, followed by CLNM. The area under the ROC curves (AUCs) for the training and validation groups were 0.849 (95% CI, 0.804–0.893) and 0.786 (95% CI, 0.667–0.905), respectively (**Figure 3**).
- Eight risk factors were included in the nomogram (**Figure 2B**) designed using clinical + US features: gender, age, PTC location, bilaterality, shape, US-ETE, nodular goiter, and US-CLNM. The US-ETE contributed the most to the prediction model, followed by full PTC. The AUCs for the training and validation groups were 0.897 (95% CI, 0.860–0.934) and 0.868 (95% CI, 0.793–0.943), respectively (**Figure 3**).
- Eight risk factors were included in the nomogram (**Figure 2C**) designed using clinical + US + pathological features: gender, age, PTC location, bilaterality, shape, US-ETE, nodular goiter, and CLNM. US-ETE contributed the most to the prediction model, followed by filled PTC. The AUCs for the training and validation groups were 0.896 (95% CI, 0.860–0.932) and 0.877 (95% CI, 0.786–0.967), respectively (**Figure 3**).

Of note, “PTC located in the lower” and “age ≥45 years “contributed least to the model in all three models.

**Figure 4:**
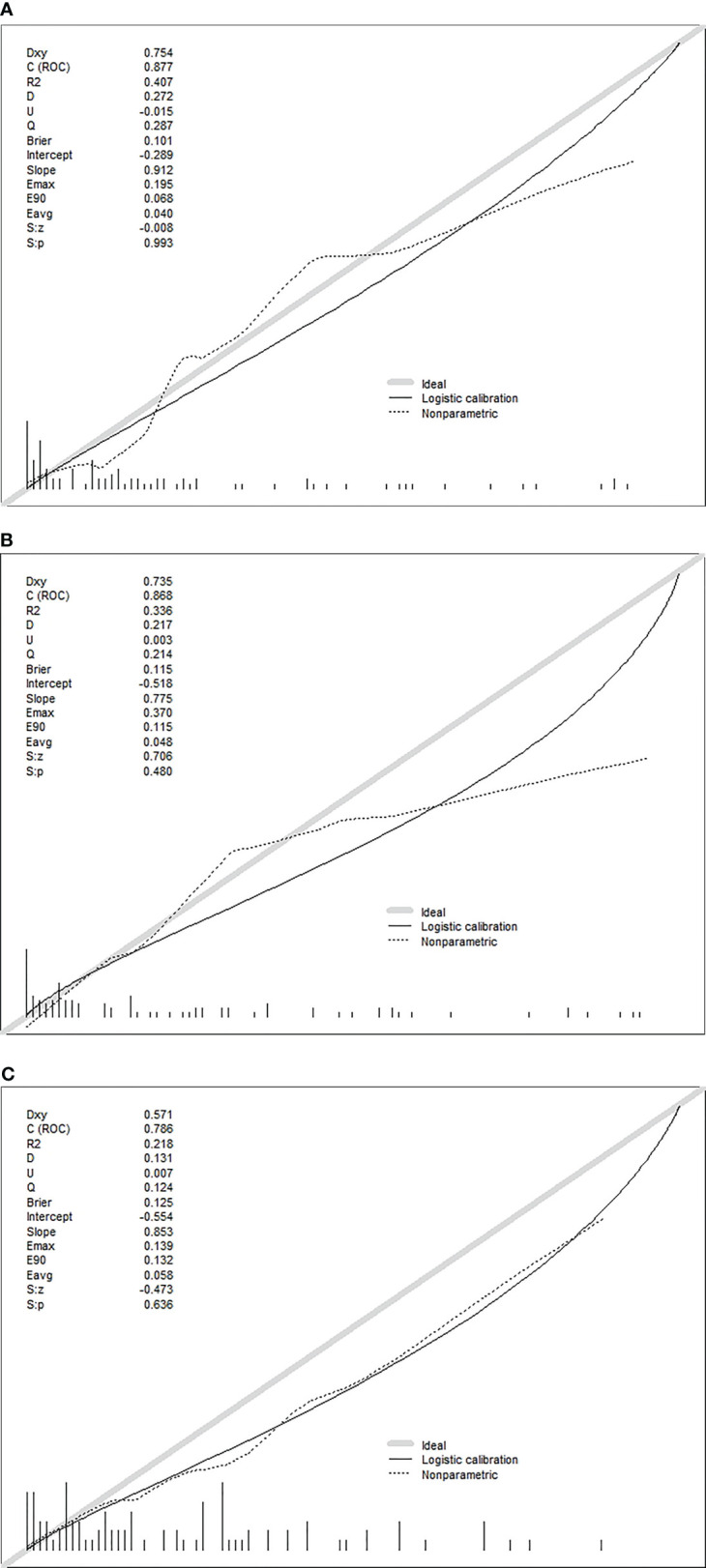
**(A)** Calibration plots of recalibrated prognostic model using the clinical + pathological features to predict the risk of DLN metastasis. **(B)** Calibration plots of recalibrated prognostic model using the clinical + US features to predict the risk of DLN metastasis. **(C)** Calibration plots of recalibrated prognostic model using the clinical + US + pathological features to predict the risk of DLN metastasis.

The calibration plots show excellent consistency of the three prediction models between the predicted metastasis probability and the actual metastasis probability (clinical + pathology, *p* = 0.636, clinical + US, *p* = 0.480, clinical + US + pathology, *p* = 0.993) (**Figure 4**). The diagnostic efficacy of the clinical + US + pathology diagnostic model was superior to that of the clinical + pathology diagnostic model (*p* = 0.039). No significant difference was reported between the clinical + US diagnostic model and the clinical + US + pathology diagnostic model (*p* = 0.724).

**Figure 5:**
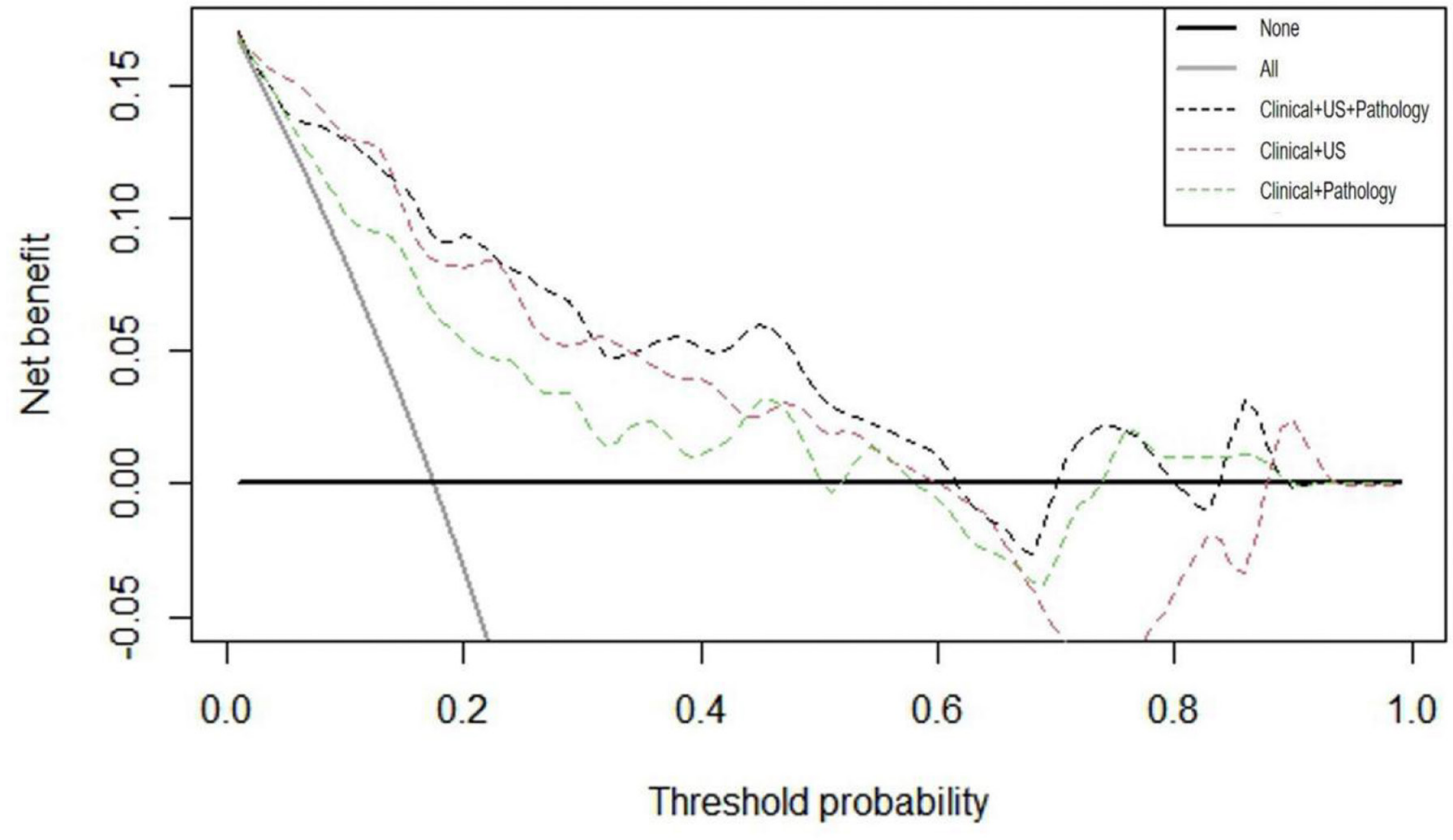
Decision curve analysis (DCA) curves of the three models.

After verifying the accuracy of the models, DCA curves were applied to the validation cohort. As illustrated in **Figure 5**, the threshold probability ranges of net benefit above the extreme curves for each model were approximated as follows: clinical + pathology 0%–50%, 52%–58%, and 74%–90% (sum 76%); clinical + US 0%–60% and 88%–93% (sum 65%); clinical + US + pathology 0%–62%, 70%–80%, and 84%–90% (sum 78%). These data provide strong evidence that all three prediction models have a high clinical efficacy and application value. If the threshold probability > 15%, application of clinical + US + pathology predicted more benefit for DLNM than treating all patients or not treating all patients, clinical + pathology, and clinical + US models.

## Discussion

Mounting evidence indicates that PTC is prone to early lymph node metastases and 30%–80% of patients are approximated to develop cervical lymph node metastases. Preoperative imaging has a probability of missing cervical lymph node metastases. In a previous study, cN0 patients were reported to have a 53.71% probability of having CLNM (16). Delphian lymph node metastasis is an independent predictor of more frequent involvement of cervical lymph nodes in many head and neck cancers and poorer patient prognosis (6, 7). Although the correlation between DLNM and the prognosis of thyroid malignancies has not been validated by relevant guidelines, previous evidence indicates that DLNM patients are 5 to 8 times more likely to develop CLNM and 3.5 times more likely to present with LLNM (8, 10, 11).

Consistent with previous reports, the present study revealed the following: (i) the incidence of CLNM in DLNM+ patients was approximately twice that of DLNM- patients; (ii) the incidence of LLNM in DLNM+ patients was approximately 1.5 times that of DLNM- patients; (iii) the incidence of DLNM+ in CLNM+ patients was three times that of CLNM- patients; and (iv) the incidence of DLNM+ in LLNM+ patients was twice that of LLNM- patients. Moreover, more CLNM and LLNM metastatic lymph nodes were reported in the DLNM+ group than in the DLNM- group. Regarding tumor aggressiveness, the incidence of T3b–T4b was approximately three times higher in DLNM+ patients than in DLNM- patients, and the incidence of T3b–T4b was 2.5 times higher than that of T1–T3a in DLNM+ patients. Four of the six patients with distant metastases had DLNM, whereas two did not have DLNM. In view of these findings, DLNM may predict whether PTC is more aggressive and is characterized by extensive lymph node metastasis. Huang et al. suggested that DLN could be used as a first step approach to determine the appropriate extent of lymph node dissection before thyroidectomy with routine intraoperative frozen section consultation (13). The findings strongly suggest that DLNM prediction would provide a better reference for clinicians’ treatment plan.

Studies found that the incidence of DLNM was 19.4%–24.5% at the time of DLN detection, which is in agreement with our finding (20.2%) (8, 9, 17). Several lines of evidence indicate that DLN metastasis is associated with gender, age, bilaterality, P-ETE, and central and lateral cervical lymph node metastasis in patients with PTC (9–11, 17–19). Some of these clinical factors have been revealed as independent risk factors for DLNM in the present study, particularly males, age <45 years, P-ETE, bilaterality, and central lymph node metastasis. Moreover, tumor location in the isthmus was an independent risk factor for DLNM. Among US features, independent risk factors for DLNM (including US-ETE, irregular tumor shape, and US-CLNM) and independent protective factors for DLNM (including tumor location in the lower pole and combined nodular goiter) were revealed.

Compelling evidence demonstrates that the majority of anterior laryngeal lymph node drainage originates from the superior thyroid lobe, the isthmus, and the conus of the thyroid. As such, anterior laryngeal lymph node metastasis is hypothesized to have a close association with the lesion location, especially in the isthmus and upper third of the thyroid gland (15, 18, 20). For instance, Chai et al. demonstrated a significant association between PTC located in the isthmus or upper third with DLNM (18). However, other studies have provided evidence that location factors are not independent risk factors for tumor detection of DLN metastases (13–15, 21, 22). In our study, the isthmus was an independent risk factor for DLNM, while the inferior thyroid pole was a protective factor for DLNM. In all three models, the inferior pole had the lowest scores, full-filled tumors had the highest scores, and tumors in other locations had close scores. These data strongly demonstrate that tumors located in the lower pole of the thyroid are less likely to develop DLNM than those in other locations.

Some US factors were of importance in the present study. For example, the irregular shape is a characteristic of highly aggressive tumors, serving as a crucial US feature and an independent risk factor for DLNM. Regarding ETE, numerous studies revealed that ETE is an independent risk factor for DLNM; however, these reports were on a pathological basis (13, 17–19, 22, 23). Notably, the present work explored both US and pathological approaches. Univariate analysis revealed a significant association of DLNM with both US-ETE and P-ETE (all *p* < 0.001). In the clinical + pathology group, P-ETE was an independent risk factor. However, in the clinical + pathology + US and clinical + US groups, where both US-ETE and P-ETE were present, only US-ETE was an independent risk factor for DLNM. Furthermore, P-ETE was about 80 points in nomogram I, while US-ETE was about 100 points in both nomograms II and III. With US, the relationship between the location of the tumor and the envelope is evident. On the other hand, it becomes challenging to determine whether the tumor invades the envelope in the imaging picture, especially when the thyroid envelope is in contact with the lesion without significant breakthrough. Nevertheless, US provides information about tumor location and the thyroid envelope, and perhaps the proximity and contact with the envelope also contribute to DLN metastasis. The 2015 American Thyroid Association (ATA) guidelines recommend US for preoperative examination of cervical lymph nodes in PTC patients (24). In the present study, US-CLNM demonstrated high specificity (95.2%), low sensitivity (34.3%), and the accuracy was 66.0%, which concur with findings of Sun et al. (25), who found that US-CLNM had high specificity (84.5%), low sensitivity (40.3%), and the accuracy was 67.7%. These data demonstrate that US has a promising preoperative predictive ability for CLNM diagnosis, which, although is not satisfactory in sensitivity, shows excellent specificity. US-CLNM was found to be an independent risk factor for the clinical + US group. However, when both US-CLNM and CLNM were present, only CLNM was an independent risk factor.

Finally, 3 different nomograms were developed based on the predictor variables identified in the multiple regression models in clinical + pathology, clinical + US, and clinical + pathology + US, respectively. The diagnostic performance of the nomograms was then compared. To our knowledge, this is the first retrospective study to explore clinical, US, and pathological risk factors in PTC patients and develop a diagnostic model to predict DLNM. The AUC of our clinical + pathology model (0.786) was similar to that of the model by Li et al. (22) (AUC 0.745). Compared with that, the clinical + pathology + US model and the clinical + US model demonstrated superior ability to predict DLNM (AUC was 0.877 and 0.868, respectively). Furthermore, DCA showed that the clinical + pathology + US model can achieve more net benefit within more threshold probabilities compared to the clinical + US model. These observations provide strong evidence that better predictive outcomes can be obtained using the clinical + pathology + US model. However, the clinical + US model, which does not rely on pathological findings, holds great promise as a useful predictive tool for designing preoperative surgical plans.

Admittedly, despite our attempts to obtain more realistic validation data by making the data from the modeling and validation groups completely independent, this study is inherently limited by a retrospective single-center design that may be subject to selection bias. Therefore, to validate these findings, further studies involving larger sample sizes and evaluation of external data sets are warranted. Secondly, ultrasonography inevitably suffers from the examiner’s subjectivity. In addition, long-term postoperative follow-up was lacking; as such, the relationship between DLN and prognosis in patients with PTC remains to be further explored.

## Conclusions

In conclusion, this study found that DLNM may be a sign that PTC is more invasive and has extensive lymph node metastasis. By exploring the clinical, pathology, and US characteristics of PTC progression to DLNM, three prediction nomograms, established according to different combinations of features, can be used in different situations to evaluate the transfer risk of DLN.

## Data Availability Statement

The raw data supporting the conclusions of this article will be made available by the authors, without undue reservation.

## Ethics Statement

Ethical review and approval was not required for the study on human participants in accordance with the local legislation and institutional requirements. Written informed consent for participation was not required for this study in accordance with the national legislation and the institutional requirements.

## Author Contributions

Conception and design: QQ and PX. Administrative support: AZ. Provision of study materials or patients: QQ, AZ and PX. Collection and assembly of data: PX, CZ, SG, XH, SC, and YL. Data analysis and interpretation: QQ and XH. Manuscript writing: All authors. All authors contributed to the article and approved the submitted version.

## Funding

This study was supported by the Key Research and Development Program of Jiangxi Province (20181BBG70031), the Postgraduate Innovation Special Fund Project of Jiangxi Province (YC2021-B043), and the Interdisciplinary Innovation Fund of Natural Science, Nanchang University (9167-28220007-YB2110).

## Conflict of Interest

The authors declare that the research was conducted in the absence of any commercial or financial relationships that could be construed as a potential conflict of interest.

## Publisher’s Note

All claims expressed in this article are solely those of the authors and do not necessarily represent those of their affiliated organizations, or those of the publisher, the editors and the reviewers. Any product that may be evaluated in this article, or claim that may be made by its manufacturer, is not guaranteed or endorsed by the publisher.
