# Supplementary material for: Nomograms Combining Ultrasonic Features With Clinical and Pathological Features for Estimation of Delphian Lymph Node Metastasis Risk in Papillary Thyroid Carcinoma
Source: Front Oncol. 2021 Dec 23;11:792347. doi: 10.3389/fonc.2021.792347 (PMC8733604; doi:10.3389/fonc.2021.792347)
Supplement: Supplementary file 1 [file DataSheet_1.docx]

Supplementary Material

A


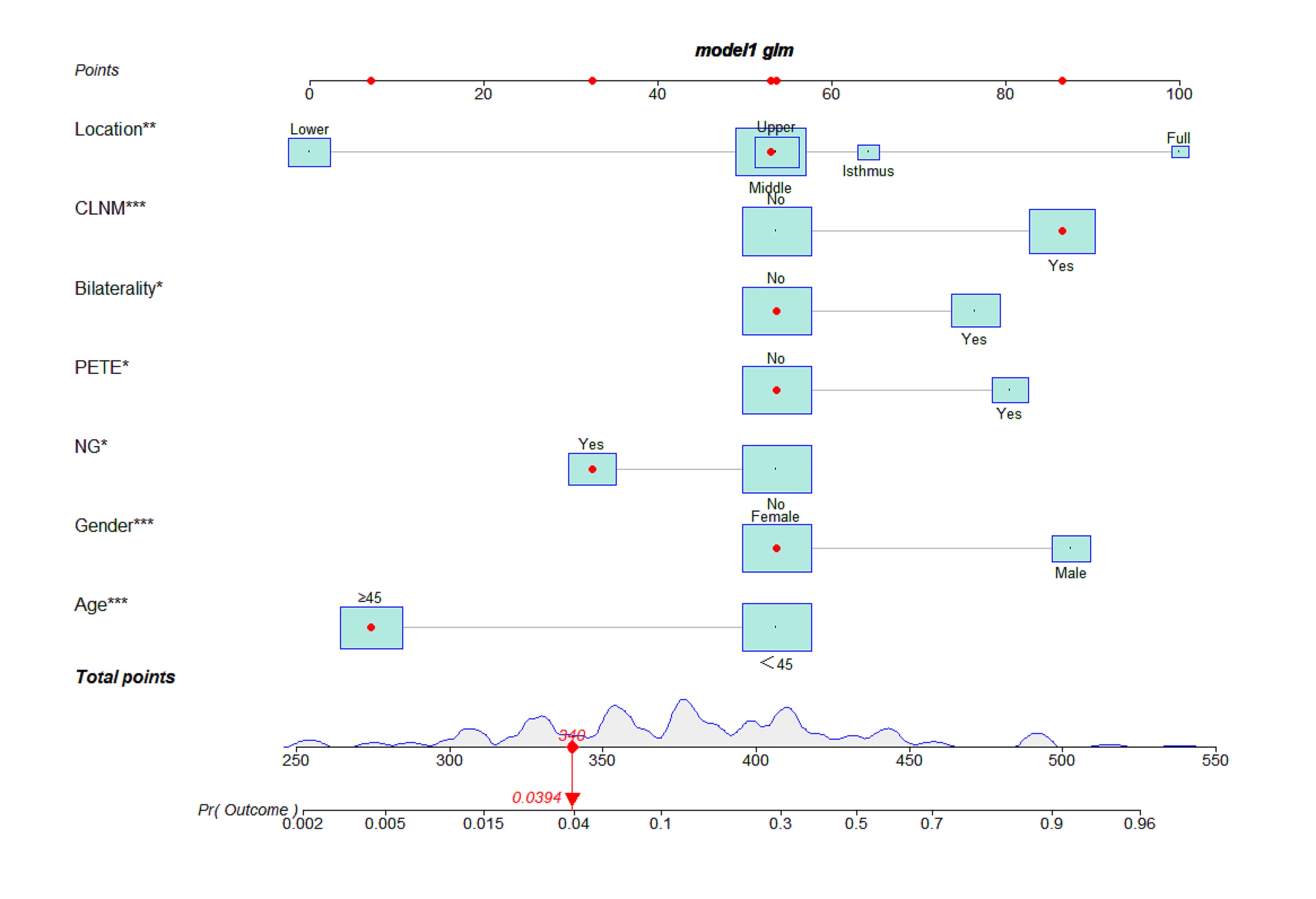


**Figure 2** **(A)**Interactive nomogram for predicting DLNM based on clinical + pathological features**.**

Score of features:Location(lower,0; middle,53;upper,54;isthmus,63;full,100 ),

CLNM(no,53;yes,87)

Bilaterality(no,53;yes,77)

1. ETE(no,53;yes,80)

Nodular goiter(no,53;yes,32)

Gender(female,53;male,88)

Age(≥45,7;＜45,53)

B


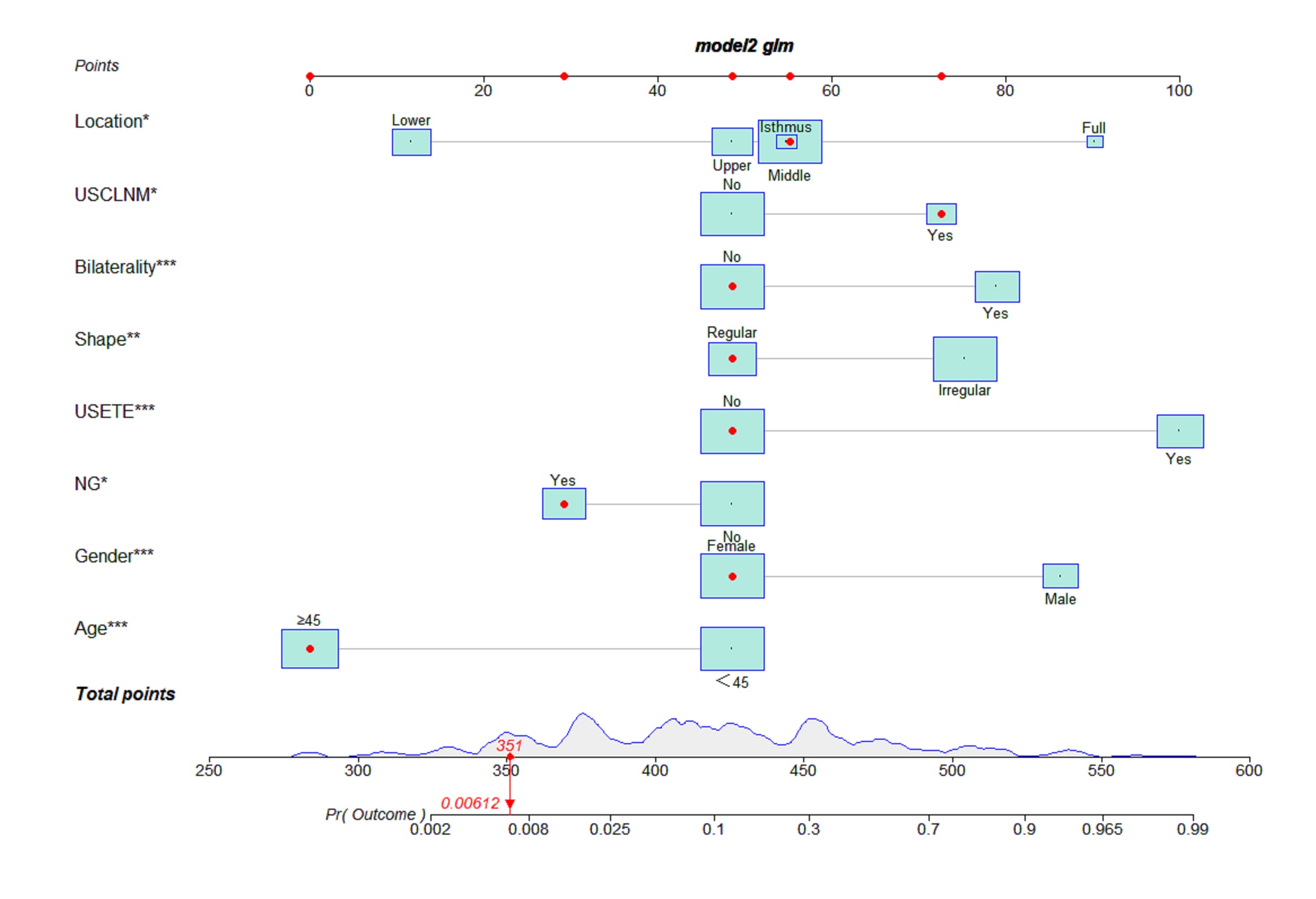


**Figure 2** **(B)**Interactive nomogram for predicting DLNM based on clinical +US features.

Score of features:Location(lower,6; upper,48,middle,55;isthmus,56;full,90)

US-CLNM(no,48;yes,72)

Bilaterality(no,48;yes,79)

Shape(regular,48;irregular,73)

US-ETE(no,48;yes,100)

Nodular goiter(no,48;yes,29)

Gender(female,48;male,87)

Age(≥45,0;＜45,48)

C


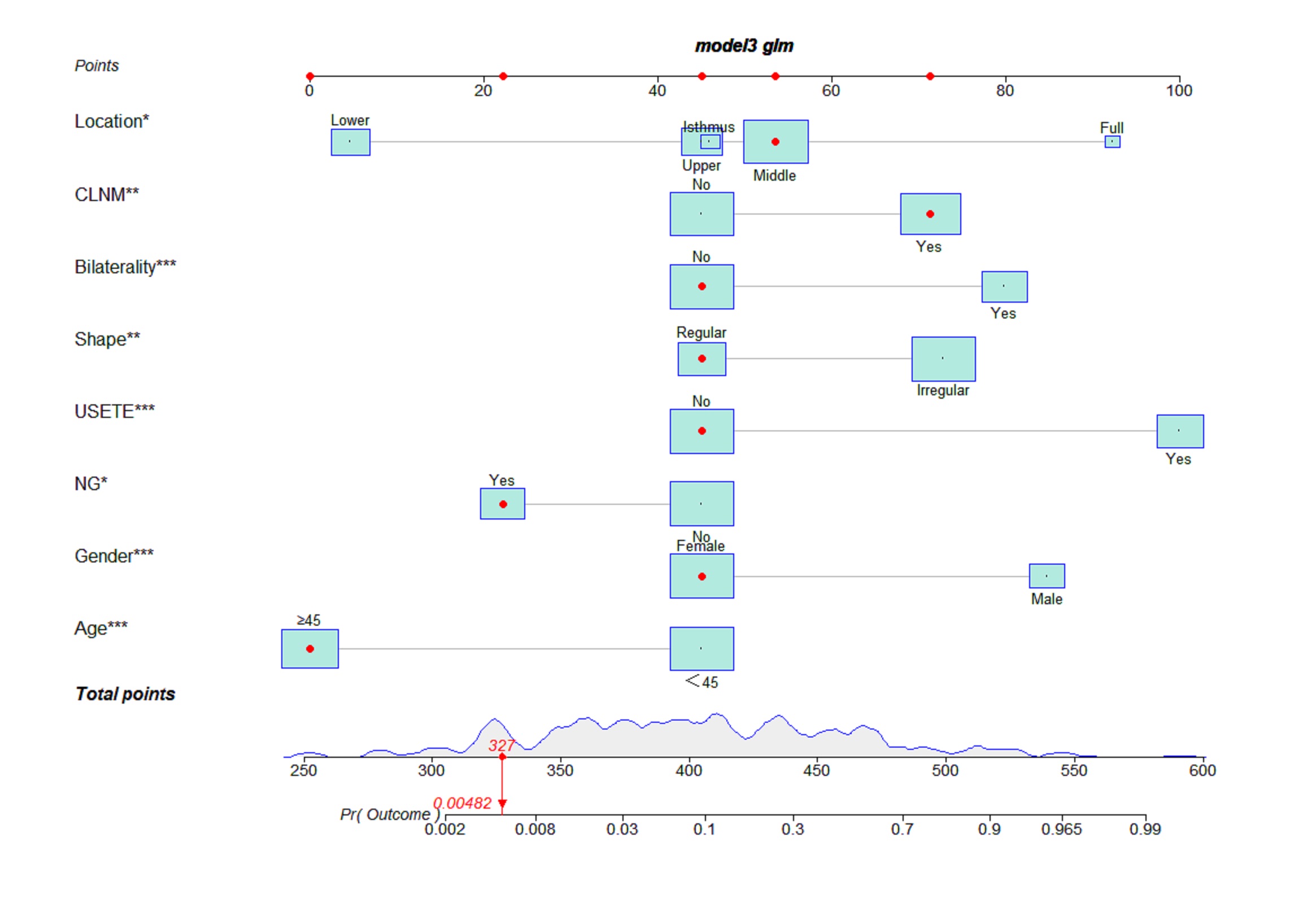


**Figure 2** **(C)**Interactive nomogram for predicting DLNM based on clinical + US+pathological features.

Score of features:Location(lower,4; upper,44,isthmus,46;middle,53;full,93)

CLNM(no,44;yes,72)

Bilaterality(no,44;yes,79)

Shape(regular,44;irregular,72)

US-ETE(no,44;yes,100)

Nodular goiter(no,44;yes,22)

Gender(female,44;male,84)

Age(≥45,0;＜45,44)
